# Machine learning and discriminant function analysis in the formulation of generic models for sex prediction using patella measurements

**DOI:** 10.1007/s00414-022-02899-7

**Published:** 2022-10-07

**Authors:** Mubarak A. Bidmos, Oladiran I. Olateju, Sabiha Latiff, Tawsifur Rahman, Muhammad E. H. Chowdhury

**Affiliations:** 1 College of Medicine, QU Health, Department of Basic Medical Sciences, Qatar University, Doha, Qatar; 2 School of Anatomical Sciences, Faculty of Health Sciences, University of the Witwatersrand, Johannesburg, South Africa; 3 Department of Electrical Engineering, College of Engineering, Qatar University, Doha, Qatar

**Keywords:** Forensic anthropology, Sex prediction, Patella, Discriminant function analyses, Machine learning

## Abstract

Sex prediction from bone measurements that display sexual dimorphism is one of the most important aspects of forensic anthropology. Some bones like the skull and pelvis display distinct morphological traits that are based on shape. These morphological traits which are sexually dimorphic across different population groups have been shown to provide an acceptably high degree of accuracy in the prediction of sex. A sample of 100 patella of Mixed Ancestry South Africans (MASA) was collected from the Dart collection. Six parameters: maximum height (maxh), maximum breadth (maxw), maximum thickness (maxt), the height of articular facet (haf), lateral articular facet breadth (lafb), and medial articular facet breath (mafb) were used in this study. Stepwise and direct discriminant function analyses were performed for measurements that exhibited significant differences between male and female mean measurements, and the “leave-one-out” approach was used for validation. Moreover, we have used eight classical machine learning techniques along with feature ranking techniques to identify the best feature combinations for sex prediction. A stacking machine learning technique was trained and validated to classify the sex of the subject. Here, we have used the top performing three ML classifiers as base learners and the predictions of these models were used as inputs to different machine learning classifiers as meta learners to make the final decision. The measurements of the patella of South Africans are sexually dimorphic and this observation is consistent with previous studies on the patella of different countries. The range of average accuracies obtained for pooled multivariate discriminant function equations is 81.9–84.2%, while the stacking ML technique provides 90.8% accuracy which compares well with those presented for previous studies in other parts of the world. In conclusion, the models proposed in this study from measurements of the patella of different population groups in South Africa are useful resent with reasonably high average accuracies.

## Introduction

Prediction of sex from recovered or discovered bones in human identification is an important first step taken by forensic anthropologists to reduce the number of possible matches by 50% [1]. This process, in conjunction with the estimation of age, stature and population affinity, is essential in the establishment of the identity of an individual from skeletons. Some bones like the skull and pelvis display distinct morphological traits that are based on shape. These morphological traits which are sexually dimorphic across different population groups have been shown to provide an acceptably high degree of accuracy in the estimation of sex [2]. While most earlier researchers and some lately [3] have focused on the use of description of the observed morphological traits, considered to be a subjective method which requires many years of experience, attention has been shifted recently to the quantification of the differences in shape that are observed on bones. These quantifications can be performed objectively using various morphometric techniques such as including and not limited to geometric morphometrics [4–11].

In the absence of the pelvis and the skull which display obvious morphological differences between males and females, size differences which are present in most bones of the postcranial skeleton can also be used for sex prediction. This metrical approach can also be used on incomplete or fragmentary remains. Standard measured parameters of different bones of the skeleton which can be easily reproducible have been analyzed through the use of various statistical methods including and not limited to logistic regression and discriminant function in different population groups. It is a well-established fact that these equations are population-specific and as such should be limited in their application to only population groups for which they were formulated to obtain acceptably high classification average accuracies. This has led to the generation of population-specific discriminant function and logistic regression equations for measurements of the skull [12–17], bones of the vertebral column [18, 19], pelvis [20–22], long bones of the upper [23–29] and lower extremities [30–36], and hand and foot bones [37–40] in different population groups of the world with acceptably high classification rates.

Similar population-specific local standards have also been established in South Africa for the prediction of sex from dimensions of the skull [37, 38]) and postcranial bones [39–46]. Most of these equations have been derived from data collected from samples of bones of South Africans of European and African descent, which are housed mainly in the Raymond Dart collection of human skeletons [47], Pretoria bone collection [48], and UCT osteological collection [49]. Recently, successful attempts have also been made to formulate population-specific equations for Mixed-Ancestry South Africans or colored [43, 50–52]. While discriminant function analysis has been widely used in sex estimation using various bones of the human skeleton in South Africa, no previous attempts have utilized other novel techniques such as machine learning algorithm for that purpose.

Over the past decade, machine learning (ML) algorithms have become increasingly integrated into clinical predictive modeling, e.g., in prognostic models using health data [53–55]. Recent reviews have also highlighted the high interest in ML approaches for clinical guidance, as well as the necessity for more prognostic studies [56]. While there is a significant rise in interest in ML in health care, only a few studies have evaluated its capability of outperforming conventional statistical models (CSMs) in terms of predictability or not. ML rapidly examines continuously expanding datasets and enables the identification of patterns and trends that may not be directly visible to clinicians [57]. Other advantages of ML are its flexibility, it is nonparametric, requires no data model for the probability distribution of the outcome variable, requires no pre‐specification of covariates, and it can process large input variables simultaneously [58, 59]. In a clinical context of predicting mortality from gastrointestinal bleeding, a systematic review demonstrated higher c‐indices and predictive capacity of ML than clinical risk scores [60]. Another study aimed at predicting bleeding risk following percutaneous coronary intervention found that ML characterized bleeding risk better than a standard discriminant analysis model [61]. Likewise, ML vs CSMs using the TOPCAT trial dataset showed that ML methods presented higher c‐indices than CSMs for readmission (0.76 vs 0.73) and predicting mortality (0.72 vs 0.66) [62].

Osteometric variations between population groups have necessitated the need to propose population specificity standards for human identification. In addition, each of these groups exhibits and expresses sexual dimorphism to various degrees. Some authors have observed major flaws in the development and application of population-specific standards for the prediction of sex [63] and stature [64, 65] leading to the proposal and recommendation for use of generic equations. This study thus aims to (1) formulate generic models for sex prediction using measurements of the patella of South Africans of African (SAAD) and European descent (SAED), as well as Mixed Ancestry South Africans (MASA) (2), and compare the classification rates obtained from the generic models using linear discriminant analysis with those obtained from machine learning algorithms.

## Materials and methods

**Fig. 1:**
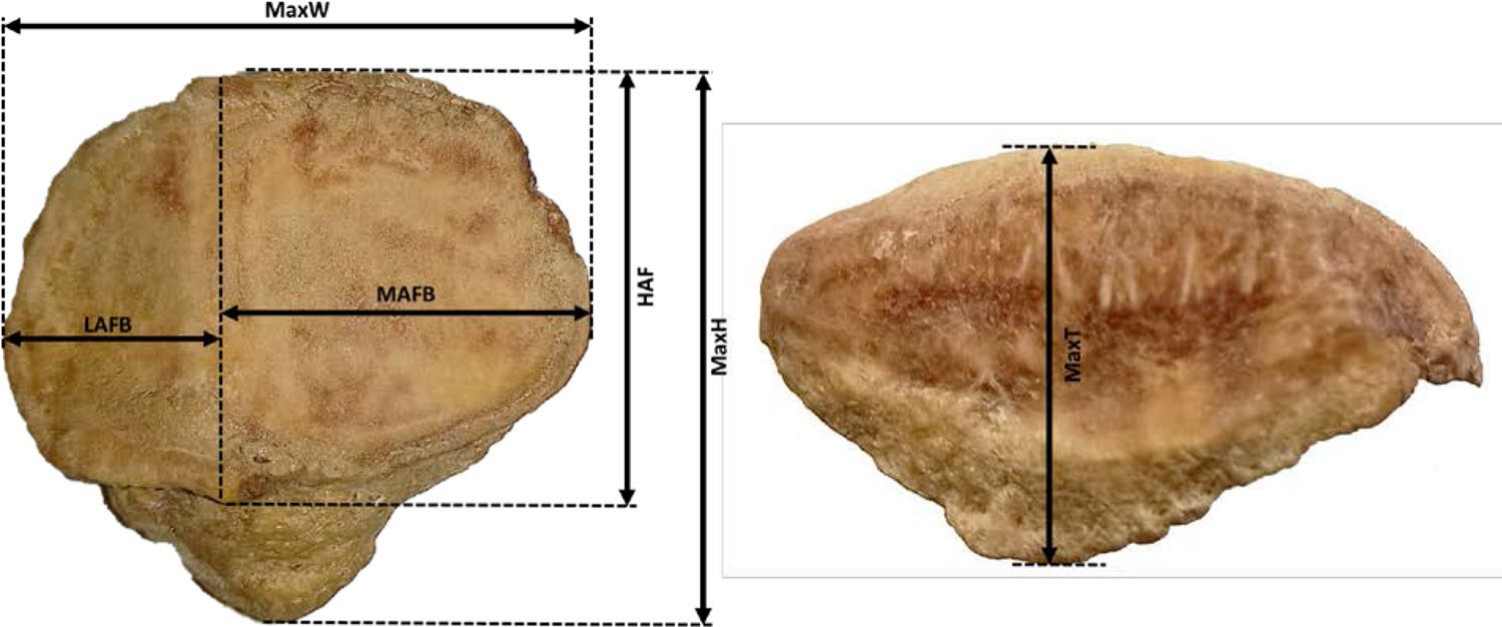
Illustration of measurements of the patella

The Human Research Ethics Committee (Medical) of the University of the Witwatersrand, Johannesburg, South Africa granted an ethical clearance waiver (Ethics Waiver Number: W-CJ-140604–1) before the commencement of this study. Data were collected from a sample of patella of Mixed Ancestry South Africans (MASA). Additional data analyzed in the current study were obtained from previously published studies on sexual dimorphism of measurements of patella of South Africans of European descent (SAED) [46] and South Africans of African descent (SAAD) [66]. The sample distribution for the data used in the current study is as follows: SAAD (50 males and 50 females), SAED (50 males and 50 females), and MASA (30 males and 30 females). The birth dates range from 1999 to 2017. The source of data was the Raymond A. Dart collection of human skeletons, considered one of the largest collections of human skeletons in the world [47]. It is located in the School of Anatomical Sciences of the University of the Witwatersrand, Johannesburg, South Africa. The patella belonged to individuals whose age at death ranged between 25 and 79 years and whose birth years were between 1928 and 1991. Patella with any pathological features like osteophytic lipping, lesions, or any other obvious deformities were excluded from this study. Six parameters were measured on each patella. These are maximum height (maxh), maximum breadth (maxw), maximum thickness (maxt), the height of articular facet (haf), lateral articular facet breadth (lafb), and medial articular facet breath (mafb). These measurements have been described in previous studies [46] and are illustrated in Fig. 1. Lin’s [67] concordance correlation coefficient of reproducibility was used for the assessment of intraobserver error. It has been shown that this method assesses the agreement between the test and retest measurement and is considered as a measure of prevision of the measuring technique.

## Statistical analysis

Statistical analysis was performed using the Stata/MP 13.0 software. SPSS version 23 software program was used for the linear discriminant analysis of data. Sex differences were described using numbers and percentages. The number of missing data, mean, standard deviations, median, and quartiles (Q1, Q3) for combined data for all measurements from SAED, SAAD, and MASA were calculated separately for each sex. In univariate analysis, the Rank sum tests were used and performed for all variables. A statistically significant difference was defined as a *P* value < 0.05.

### Discriminant function analysis

Stepwise and direct discriminant function analyses were performed for measurements that exhibited significant sex differences. The “leave-one-out” classification procedure was then used to evaluate the validity of the functions. In this procedure, each case in the sample is classified using the function that is generated without it. Then, generic stepwise and direct discriminant functions with acceptably high average accuracies were selected. Each of the generic functions selected was used to predict sex for each case in samples of SAED, SAAD, and MASA. The average accuracies in correct sex prediction for each of the functions were calculated for each population group separately.

### Machine learning-based analysis

**Fig. 2:**
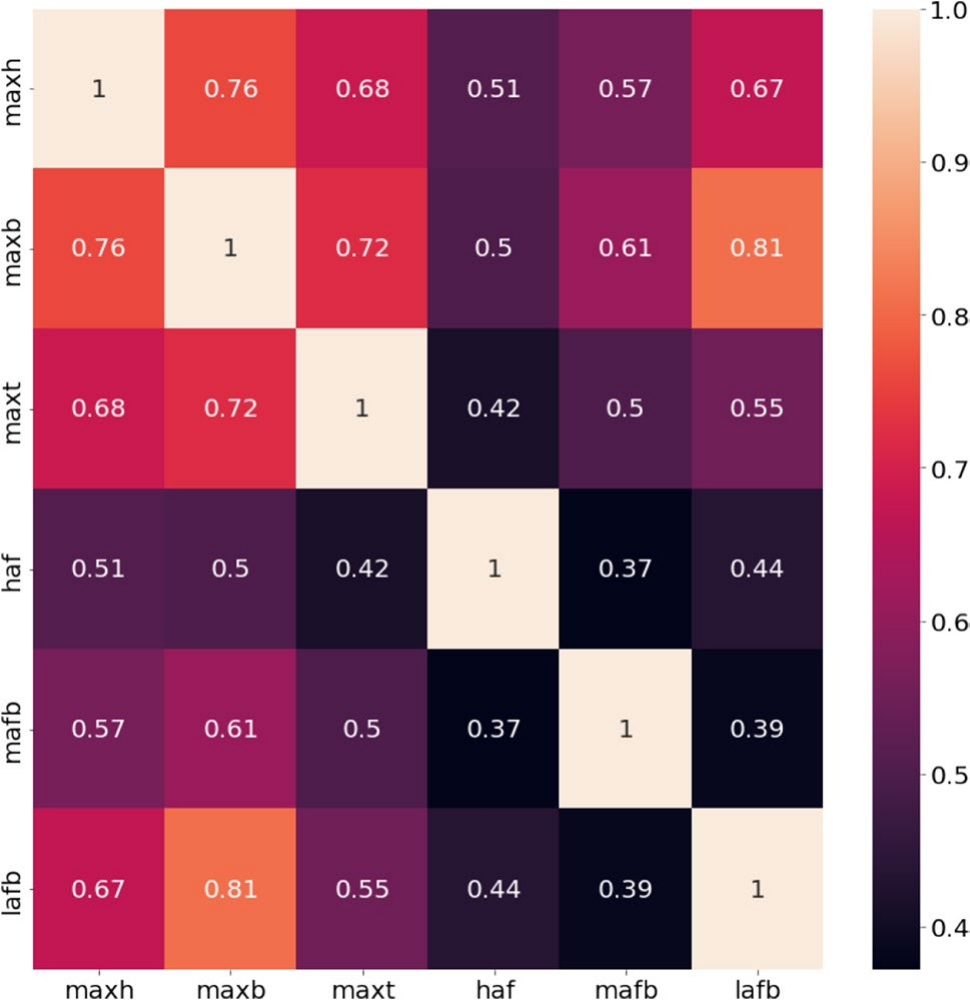
Heatmap of Pearson correlation among different features

Six patella measurements were present in the dataset that were evaluated to determine the Pearson correlation among them. Figure 2 shows the heatmap of correlation, and it was found that none of them is highly correlated to the other. A maximum correlation of 0.81 was found between maxb and lafb. However, the threshold of removing highly correlated features was considered *r* > 0.85 and therefore, none of the features was removed for the next phase of the investigation.

#### Data normalization

The accuracy of the machine learning models is dependent on the quality of the input data for achieving generalized performance. This involves data normalization that entails scaling or transforming the data to make each selection contribute equally during the training process. The performance enhancement of the machine learning models employing such has been verified by many studies [68]. In this study, *Z*-score normalization was utilized due to its sensitivity to outliers. The formula for *Z*-score normalization as shown in Eq. (1) is:

1 $${\mathrm v}^{^{\prime}}=\frac{\mathrm v-{\mathrm \mu }_{\mathrm v}}{{\mathrm \sigma }_{\mathrm v}}$$

where ${v}^{^{\prime}}$, $v$, ${\mu }_{v}$, and ${\sigma }_{v}$ denote the new value, original value, mean, and standard deviation of the variable values in the training samples, respectively. This method transforms the data with a mean of 0 and a standard deviation of 1.

#### Top-ranked features identification

The feature selection technique automatically selects those features which are most significant for output prediction. This method thus helps in reducing overfitting and training time as well as improving accuracy. Several different feature selection techniques, e.g., univariate selection, recursive feature elimination (RFE), principal component analysis (PCA), bagged decision trees like random forest and extra trees, and boosted trees like Extreme Gradient Boosting (XGBoost) etc. have been used in the literature. However, the present study investigated and compared three feature selection techniques: (1) XGBoost [69], (2) Extra tree [70], and (3) Random Forest [71, 72] to determine the best feature combinations for sex prediction using different ML classifiers.

#### Model development

The present used and compared different machine learning classifiers such as Gradient boosting [69], XGBoost [73], Extra tree [73], K-nearest neighbour (KNN) [73], Adaboost [73], Random Forest [73], linear discriminant analysis (LDA) [71, 72], and Logistic regression [74] using the best feature combination which was identified by the feature selection techniques for sex prediction. Then we investigated a stacking approach where a combination of base learners and meta learners was used to classify the sex of the subject. Here, we have used the top performing three ML classifiers as base learners and the predictions of these models were used as inputs to different machine learning classifiers as meta learners to make the final decision. Eight different machine learning classifiers as meta learners in the stacking approach to find the best performing classifier were investigated.

**Fig. 3:**
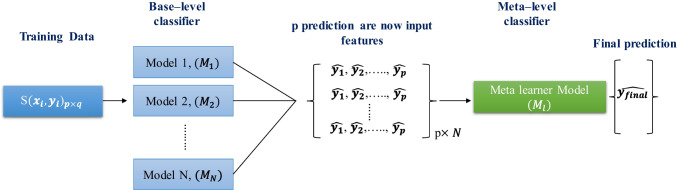
Stacking model architecture

If a single dataset A, which consists of feature vectors (${{\varvec{x}}}_{{\varvec{i}}}$) and their classification probability score is ${{\varvec{y}}}_{{\varvec{i}}}$. At first, a set of base-level classifiers ${{\varvec{M}}}_{1},\dots \dots ,{{\varvec{M}}}_{{\varvec{p}} }$ is generated and the outputs are used to train the meta-level classifier as illustrated in Fig. 3.

Five-fold cross-validation was used to create a training set for the meta-level classifier. Among these folds, base-level classifiers were trained on four-folds, leaving one fold for validation. Each base-level classifier predicts a probability distribution over the possible class values. Thus, using input *x*, a probability distribution is created using the predictions of the base-level classifier set, M:

2 $$\mathrm P^{\mathrm M}(\mathrm x)=(\mathrm P^{\mathrm M}\left({\mathrm c}_1\vert\mathrm x\right),\mathrm P^{\mathrm M}\left({\mathrm c}_2\vert\mathrm x\right),\dots\dots.,\mathrm P^{\mathrm M}\left({\mathrm c}_{\mathrm n}\vert\mathrm x\right))$$

where ${({\varvec{c}}}_{1},{{\varvec{c}}}_{2},\dots \dots ,{{\varvec{c}}}_{{\varvec{n}}})$ is the set of possible class values and ${{\varvec{P}}}^{{\varvec{M}}}\left({{\varvec{c}}}_{{\varvec{i}}}|{\varvec{x}}\right)$ denotes the probability that example *x* belongs to a class ${{\varvec{c}}}_{{\varvec{j}}}$ as estimated (and predicted) by classifier M in Eq. 2. The class, ${{\varvec{c}}}_{{\varvec{i}}}$ with the highest-class probability, ${{\varvec{P}}}^{{{\varvec{M}}}_{{\varvec{j}}}}\left({{\varvec{c}}}_{{\varvec{i}}}|{\varvec{x}}\right)$ is predicted by the classifier, M. The meta-level classifier ${{\varvec{M}}}_{{\varvec{f}}}$ and attributes are thus the probabilities predicted for each possible class by each of the base-level classifiers, i.e., ${{\varvec{P}}}^{{{\varvec{M}}}_{{\varvec{j}}}}\left({{\varvec{c}}}_{{\varvec{i}}}|{\varvec{x}}\right)$ for *i* = 1,…., *n*, and *j* = 1,…., *p*. The pseudo-code for the stacking approach is shown in Algorithm 1.

**Algorithm 1:**
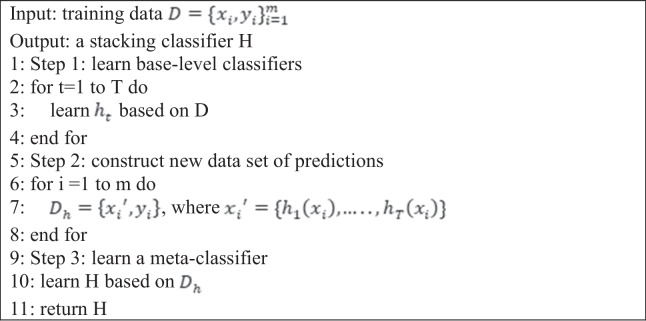
Stacking classification

#### Performance metrics

Different classification models were compared using the top-ranked features from the testing data to calculate the performance matrices in classifying male and female classes. The best performing classifier was evaluated for different combinations of features as input to the model by calculating the receiver operating characteristic (ROC)—area under the curve (AUC) and performance metrics such as accuracy, precision, sensitivity, specificity, and F1-Score as shown in Eqs. (3–7). Different classification algorithms and different features’ combinations of the best performing algorithm were validated using fivefold cross-validation where training and testing were done on 80% and 20% of data, respectively, and this process was repeated 5-times to test the entire dataset. Weighted average within 95% confidence interval was calculated for sensitivity, specificity, precision, F1-score, and overall accuracy from the confusion matrix that accumulates all test (unseen) fold results of the fivefold cross-validation. The correct estimation of a male subject is true positive (TP), and the correct estimation of the female subject is true negative (TN). The incorrect estimation of the male subject as female is false negative (FN) and the incorrect estimation of the female subject as male is false positive (FP)

3 $$\mathrm{Accuracy}=\frac{\mathrm{TP}+\mathrm{TN}}{\mathrm{TP}+\mathrm{TN}+\mathrm{FP}+\mathrm{FN}}$$

4 $$\mathrm{Precision}=\frac{\mathrm{TP}}{\mathrm{TP}+\mathrm{FP}}$$

5 $$\mathrm{Sensitivity}=\frac{\mathrm{TP}}{\mathrm{TP}+\mathrm{FN}}$$

6 $$\mathrm{Specificity}=\frac{\mathrm{TN}}{\mathrm{TN}+\mathrm{FP}}$$

7 $$\mathrm F1-\mathrm{score}=2\frac{\mathrm{Precision}\times\mathrm{Sensitivity}}{\mathrm{Precision}+\mathrm{Sensitivity}}$$

## Results

The values of Lin’s concordance correlation coefficient of reproducibility ranges between 0.974 and 0.998 (Table 1). These values fell within the recommended range from 0.90 to 0.99 which indicates that all patella measurements are easily reproducible and the subsequent data analyzed in this study are not significantly affected by measurement error. For clarity, the analyses on discriminant function and machine learning are presented separately. In the first section, results from descriptive statistics, univariate and multivariant discriminant function analysis are presented while in the second section, best feature selection, validation of ML model and stacking technique are reported.

**Table 1:** Table of concordance correlation coefficients of reproducibilty (P_c_)

Measurement	P_c_
MAXT	0.994
MAXB	0.998
MAXH	0.997
LAFB	0.994
HAF	0.974
MAFB	0998

### Discriminant function analysis

The descriptive statistics of all measurements for pooled data are shown in Table 2. The male showed significantly higher (*p* ≤ 0.05) mean measurements for all measures than the female. All patella measurements were subjected to stepwise and direct discriminant function analyses. The unstandardized coefficients, constants, average accuracies, cross-validation in correct sex classification, and the sectioning points for individual measurements are shown in Table 3. The best performing variable, maxh, presented with an acceptably high average accuracy of 82% (Table 3) while the other variables presented with low average accuracies which ranged between 69 for lafb and 79% for maxb (Table 3).

**Table 2:** Characteristics of the study subjects and univariate analysis

Item	Male	Female	Total	Method	Statistic	*P* value
Maxh • N (missing) • Mean ± SD • Median • Q1, Q3 • Min, max	130 (0) 42.07 ± 3.14 42.15 40, 44 32.49, 51	130 (0) 37.25 ± 2.8 37 35.2, 38.9 31, 52	260 (0) 39.66 ± 3.8 39 36.8, 42.5 31, 52	Rank-sum test	*Z* = − 13.11	< 0.05
Maxb • N(missing) • Mean ± SD • Median • Q1, Q3 • Min, max	130 (0) 43.77 ± 3.28 44 41.5, 46 34.84, 52	130 (0) 38.85 ± 3.23 38.9 36.82, 40.7 31.73, 51	260(0) 41.3 ± 4.1 41 38.13, 44.2 31.73, 52	Rank-sum test	*Z* = − 18.26	< 0.05
maxt • N(missing) • Mean ± SD • Median • Q1, Q3 • Min, max	130 (0) 20.38 ± 1.62 20.35 19.15, 21.5 15.7, 25.4	130 (0) 18.13 ± 1.75 18 17, 19.5 14.5, 24	260 (0) 19.26 ± 2.02 19.17 18, 20.73 14.5, 25.4	Rank-sum test	*Z* = − 9.6	< 0.05
haf • N(missing) • Mean ± SD • Median • Q1, Q3 • Min, max	130 (0) 30.26 ± 2.65 30 28.72, 32 23.3, 37	130 (0) 27.42 ± 2.8 27 25.7, 29 22, 39	260 (0) 28.85 ± 3.06 29 26.5, 30.77 22, 39	Rank-sum test	*Z* = − 11.08	< 0.05
mafb • N(missing) • Mean ± SD • Median • Q1, Q3 • Min, max	130 (0) 19.7 ± 2.02 20 18.05, 21 14.9, 24	130 (0) 17.44 ± 2.23 17.3 16, 18.47 12.3, 24.92	260 (0) 18.57 ± 2.4 18.2 17, 20.4 12.3, 24.92	Rank-sum test	*Z* = − 14.77	< 0.05
lafb • N(missing) • Mean ± SD • Median • Q1, Q3 • Min, max	130 (0) 26.34 ± 2.7 26 24.6, 28 20.8, 38	130 (0) 23.4 ± 2.6 23 21.71, 25 16.92, 32	260 (0) 24.87 ± 3.02 25 22.57, 26.98 16.92, 38	Rank-sum test	*Z* = − 11.34	< 0.05
Outcome (%)	130 (50%)	130 (50%)	260

**Table 3:** Univariate discriminant function analysis

Variable	Unstandardized coefficient	Constant	Average (O)	Average (C)
MAXH	0.336	− 13.320	81.5 (M = 79.2, F = 83.8)	81.5 (M = 79.2, F = 83.8)
MAXB	0.307	− 12.675	78.5 (M = 76.9, F = 80.0)	78.1 (M = 76.9, F = 79.2)
MAXT	0.593	− 11.420	74.6 (M = 73.1, F = 76.2)	74.6 (M = 73.1, F = 76.2)
MAFB	0.471	− 8.749	72.7 (M = 70.0, F = 75.4)	72.7 (M = 70.0, F = 75.4)
HAF	0.368	− 10.602	71.9 (M = 73.8, F = 70.0)	71.9 (M = 73.8, F = 70.0)
LAFB	0.377	− 9.374	69.2 (M = 71.5, F = 66.9)	69.2 (M = 71.5, F = 66.9)

Discriminant function equation (*y*) = unstandardized coefficient *x* variable + constant

Sectioning point is 0: *O* original classification rate before cross validation; *C* classification rate after cross validation

Table 4 shows the stepwise and direct discriminant function analysis using various combinations of measurements. In the stepwise analysis, four measurements were selected, namely, maxh, maxb, maxt, and haf. The discriminant function equation derived from these measurements provided an average accuracy of 84.2% as shown in Table 4. The other functions in Table 4 were formulated using direct discriminant function analysis of patella measurements. The average accuracies in correct sex classification ranged between 81.9 (Function D5, Table 4) and 83.5% (Function D1, Table 4). The results of the cross-validation using the leave-one-out classification showed that the average accuracy in correct sex classification for most of the presented functions remained unchanged (Table 4). Functions D2 and D4 showed a minimal and insignificant drop in classification rate of 0.8% thereby confirming the validity of the derived functions from the pooled data.

**Table 4:** Multivariate discriminant function analysis

	Variables	Unstandardized coefficient	Average accuracies (%)	
			O	C
Stepwise	maxh	0.163	84.2 (M = 83.8, F = 84.6)	84.2 (M = 83.8, F = 84.6)
	maxb	0.09		
	maxt	0.144		
	haf	0.099		
	Constant	− 15.860		
Direct				
D1	maxh	0.213	83.5 (M = 80.0, F = 86.9)	83.5 (M = 80.0, F = 86.9)
	maxb	0.146		
	Constant	− 14.476		
D2	maxh	0.188	83.5 (M = 80.8, F = 86.2)	82.7 (M = 79.2, F = 86.2)
	maxb	0.110		
	maxt	0.149		
	Constant	− 14.849		
D3	lafb	-0.021	83.1 (M = 81.5, F = 84.6)	83.1 (M = 81.5, F = 84.6)
	maxb	0.087		
	maxh	0.158		
	maxt	0.136		
	haf	0.097		
	mafb	0.066		
	Constant	− 15.969		
D4	maxh	0.248	83.1 (M = 83.1, F = 83.1)	82.3 (M = 81.5, F = 83.1)
	maxt	0.233		
	Constant	− 14.322		
D5	maxh	0.294	81.9 (M = 79.2, F = 84.6)	81.9 (M = 79.2, F = 84.6)
	lafb	0.076		
	Constant	− 13.568		

*O* original classification rate before cross validation*; C* classification rate after cross validation

### Machine learning analysis

#### Best feature combination for sex prediction

**Fig. 4:**
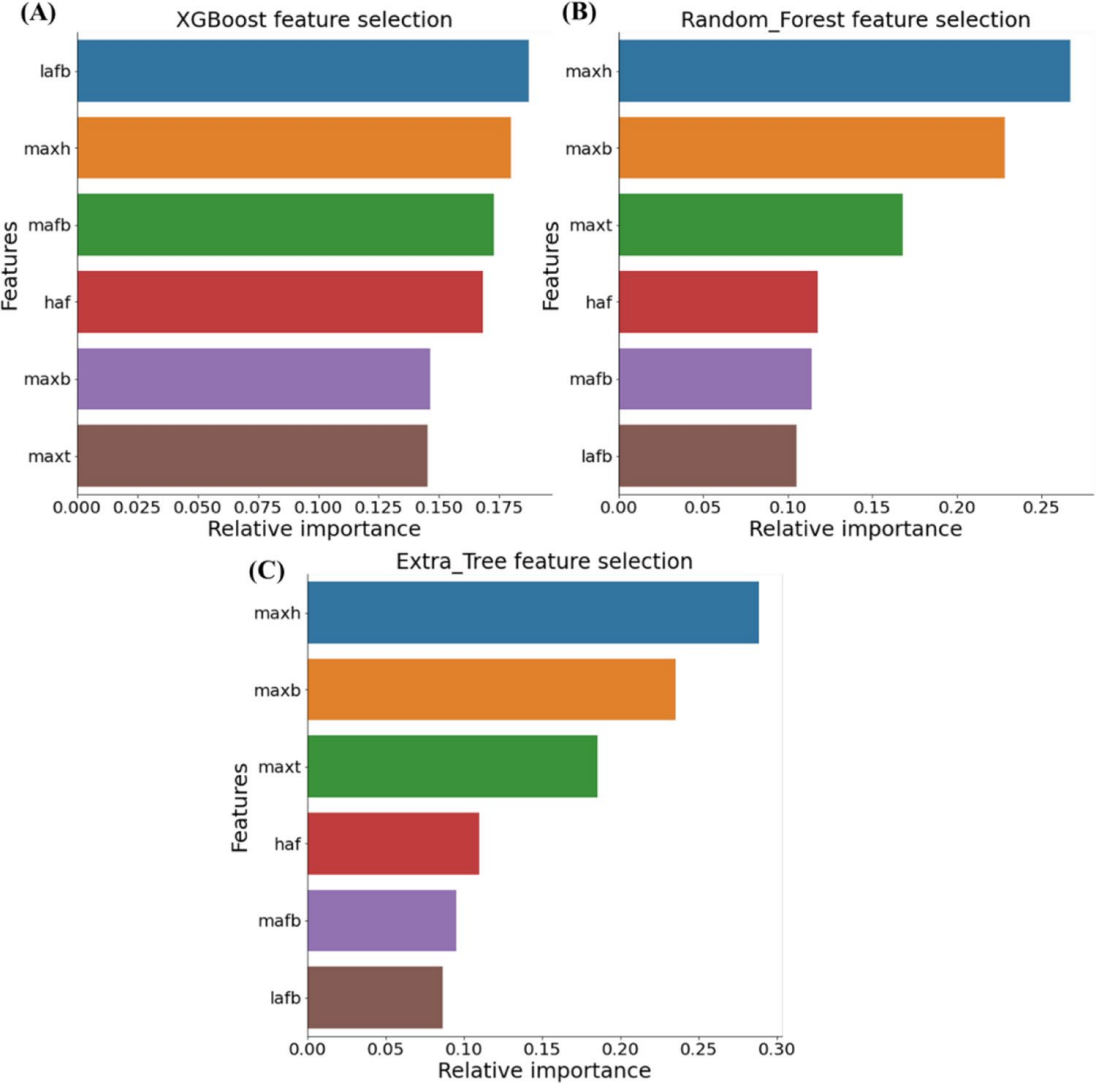
Top-ranked features using different feature selection techniques; **A** XGBoost, **B** random forest, and **C** Extra Tree algorithms

In this study, three feature ranking algorithms were used to identify top-ranked features among all features. These top-ranked features were investigated with 8 different classifiers which were performed with Top-1 to Top-6 features to identify the best performing classification model and best feature combination simultaneously for sex prediction. It was observed that RF and ET feature selection techniques produced the same feature ranking while the XGBoost feature selection algorithm produced different rankings as shown in Fig. 4.

In this study, Top-3 features (maxh, maxb, and maxt) using a random forest (RF) feature selection algorithm with random forest machine learning (ML) classifier outperformed other classifiers. Table 5 shows the overall accuracies and weighted average performance for the other matrices (precision, sensitivity, specificity, and F1-score) with a 95% confidence interval to identify the best feature combinations using Top 1 to 6 features for fivefold cross-validation using the best classifier (AdaBoost classifier for XGBoost feature selection and RF classifier for RF and ET feature selection algorithms).

**Table 5:** Comparison of the average performance metrics from five-fold cross-validation for top-ranked features using the best performing ML classifier

Feature selection	Best ML classifier	Features	Weighted figures with 95% CI				
			Overall accuracy	Precision	Sensitivity	Specificity	F1-score
XGBoost	AdaBoost	Top-1	75.01 ± 5.26	74.88 ± 5.27	75.1 ± 5.26	75 ± 5.26	74.8 ± 5.28
		Top-2	86.92 ± 4.1	86.93 ± 4.1	86.92 ± 4.1	86.92 ± 4.1	86.92 ± 4.1
		Top-3	88.08 ± 3.94	88.19 ± 3.92	88.08 ± 3.94	88.08 ± 3.94	88.07 ± 3.94
		Top-4	88.85 ± 3.83	88.87 ± 3.82	88.85 ± 3.83	88.85 ± 3.83	88.84 ± 3.83
		Top-5	87.69 ± 3.99	87.73 ± 3.99	87.69 ± 3.99	87.69 ± 3.99	87.69 ± 3.99
		**Top-6**	**89.23 ± 3.77**	**89.31 ± 3.76**	**89.23 ± 3.77**	**89.23 ± 3.77**	**89.23 ± 3.77**
RF and ET	RF	Top-1	80.77 ± 4.79	80.8 ± 4.79	80.77 ± 4.79	80.77 ± 4.79	80.76 ± 4.79
		Top-2	84.23 ± 4.43	84.28 ± 4.42	84.23 ± 4.43	84.23 ± 4.43	80.76 ± 4.79
		**Top-3**	**89.23 ± 3.77**	**88.64 ± 3.86**	**90 ± 3.65**	**88.46 ± 3.88**	**89.31 ± 3.76**
		Top-4	87.31 ± 4.05	87.33 ± 4.04	87.31 ± 4.05	87.31 ± 4.05	87.31 ± 4.05
		Top-5	88.08 ± 3.94	88.1 ± 3.94	88.08 ± 3.94	88.08 ± 3.94	88.08 ± 3.94
		Top-6	88.08 ± 3.94	88.13 ± 3.93	88.08 ± 3.94	88.08 ± 3.94	88.07 ± 3.94

It is clearly seen that the Top-3 features (maxh, maxb, and maxt) from RF and ET feature selection techniques produced the best performance of overall accuracy, and weighted precision, sensitivity, specificity, and F1-score of 89.61%, 89.67%, 89.62%, 89.62%, and 89.61%, respectively, using RF classifier for sex prediction. It was noticed that six features were required in the case of the XGBoost feature selection technique to produce the best performance of overall accuracy, weighted precision, sensitivity, specificity, and F1-score of 89.23%, 89.31%, 89.23%, 89.24%, and 89.22%, respectively, using AdaBoost classifier, whereas similar performance was produced by only three features from RF and ET feature selection techniques with RF classifier.

#### Development and validation of different ML and stacking models

We investigated the best combination of three features (maxh, maxb, and maxt) and selected the best ML classifiers among eight classifiers as base models and trained different ML classifiers as meta-learners. We selected top two models (RF and ET) where the overall accuracies, and weighted precision, sensitivity, specificity, and F1-score were 89.23%, 88.64%, 90.00%, 88.46%, 89.31%, and 85.34%, 85.27%, 85.03%, 85.45%, 85.14%, respectively (Table 6). The stacking approach was trained with RF and ET classifiers as a base learner and Gradient Boosting classifier as meta learner outperformed other meta learner classifiers with the performance of overall accuracy, and weighted precision, sensitivity, specificity, and F1-score of 90.77%, 89.55%, 92.3%, 89.23%, and 90.9%, respectively.

**Table 6:** Comparison of the average performance metrics from five-fold cross-validation for different classifiers and stacking classifiers

Classifier	Weighted with 95% CI				
	Overall accuracy	Precision	Sensitivity	Specificity	F1-score
Linear discriminant analysis	83.85 ± 4.47	83.85 ± 4.47	83.85 ± 4.47	83.85 ± 4.47	83.85 ± 4.47
XGB classifier	81.15 ± 4.75	81.2 ± 4.75	81.15 ± 4.75	81.15 ± 4.75	81.15 ± 4.75
Random forest classifier	**89.23 ± 3.77**	**88.64 ± 3.86**	**90 ± 3.65**	**88.46 ± 3.88**	**89.31 ± 3.76**
Logistic regression	83.85 ± 4.47	83.85 ± 4.47	83.85 ± 4.47	83.85 ± 4.47	83.85 ± 4.47
Extra trees classifier	**85.34 ± 4.3**	**85.27 ± 4.31**	**85.03 ± 4.34**	**85.45 ± 4.29**	**85.14 ± 4.32**
AdaBoost classifier	83.46 ± 4.52	83.51 ± 4.51	83.46 ± 4.52	83.46 ± 4.52	83.46 ± 4.52
K neighbors classifier	83.08 ± 4.56	83.27 ± 4.54	83.08 ± 4.56	83.08 ± 4.56	83.05 ± 4.56
Gradient boosting classifier	85 ± 4.34	85.1 ± 4.33	85 ± 4.34	85 ± 4.34	85.03 ± 4.34
Stacking model	**90.77 ± 3.52**	**89.55 ± 3.72**	**92.3 ± 3.24**	**89.23 ± 3.77**	**90.9 ± 3.5**

**Fig. 5:**
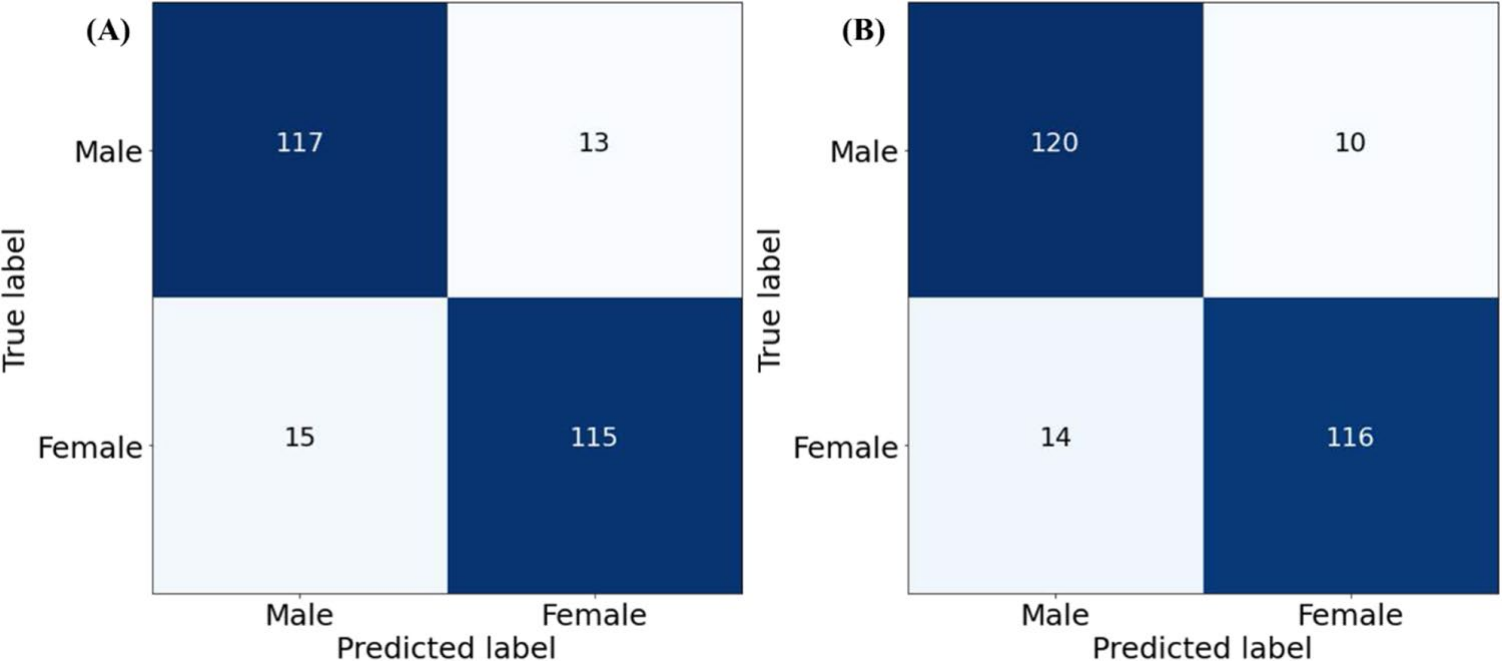
Confusion matrix for sex prediction model using **A** random forest classifier and **B** stacking classifier

Figure 5A shows the confusion matrix of the best performing ML classifier (RF classifier), and Fig. 5B shows the confusion matrix of the best performing stacking model (with Gradient Boosting classifiers as a meta learner). It can be noticed that even with the best performing RF classifier, 13 out of 130 male subjects were miss-classified as female and 15 out of 130 female subjects were miss-classified as male when the stacking model with Gradient Boosting classifier as a meta learner outperformed other ML classifiers, where 120 out of 130 male subjects were correctly classified as male and 116 out of 130 females were correctly identified as a female with the stacking model. Thus, the stacking model outperformed other state-of-the-art ML classifiers.

**Fig. 6:**
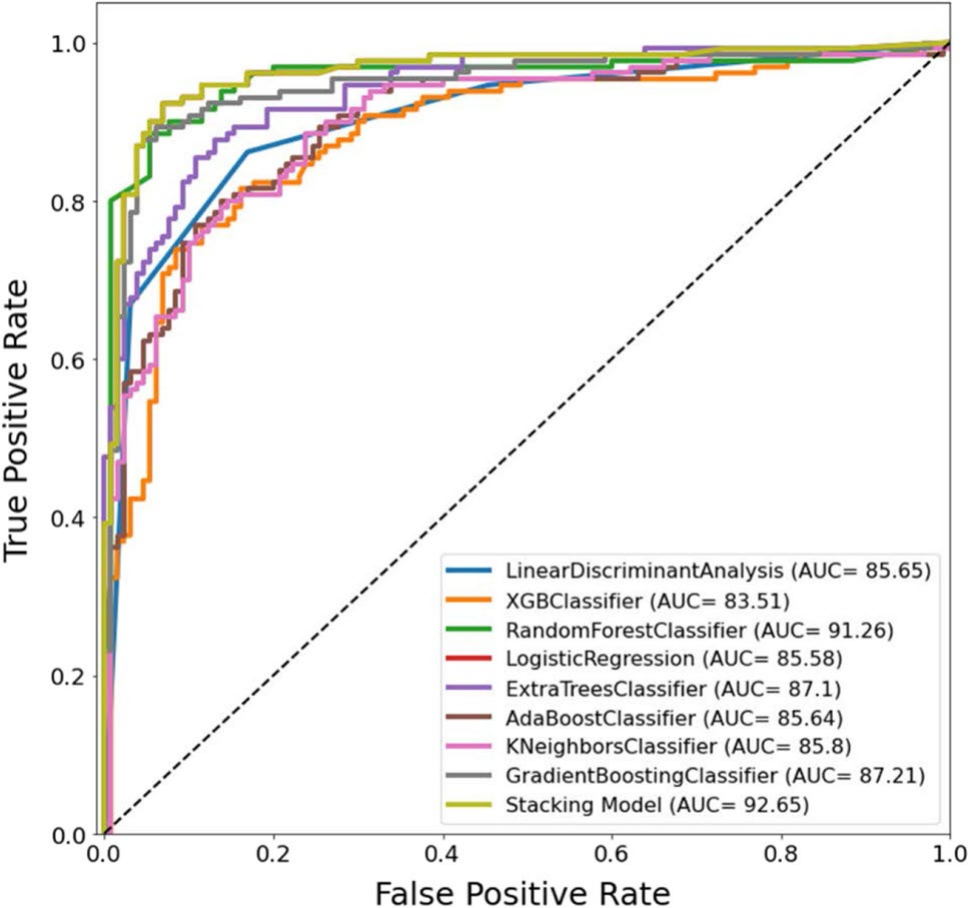
ROC curve for sex prediction classifier using different ML models and stacking classifier

Figure 6 shows the AUC) /ROC curve (also known as AUROC (area under the receiver operating characteristics)) for sex identification using different ML classifiers, which is one of the most important evaluation metrics for checking any classification model’s performance. It is apparent that the stacking model outperformed other ML classifiers for classification with 92.65% AUC (Fig. 6).

## Discussion

Sex prediction from measurements of bones that display sexual dimorphism is an important aspect of forensic anthropology. Population-specific standards which are generally considered to provide the best estimation of sex have been published for the skull and postcranial elements in different parts of the world [30, 40, 44, 50, 75, 76]. These became necessary because of the observed variation in the display of sexual dimorphism between different population groups [26]. Consequently, the application of standards for population groups is not encouraged for other groups. One disadvantage of using population-specific standards is having prior knowledge of the population group of the skeleton under forensic analysis [63, 64].

In the present study, patella measurements of South Africans were shown to be sexually dimorphic and which is consistent with the results of previous studies on patella of Italians [77], Americans [78], Iranians [79], Spaniards [80], African Americans [81], Japanese [82], Turks [83], and Swiss [84]. The range of average accuracies obtained for pooled multivariate discriminant function equations (DFEs) and stacking ML technique in the current study (81.9–90.8%, Tables 4 and 6) compares well with those presented for previous studies in other parts of the world (Table 7). It is interesting to note that the highest average accuracies for all studies that utilized skeletal collection in the acquisition of data are approximately not more than 85% [46, 66, 80, 81, 84]. Other studies in which data were collected from radiological modalities and autopsy acquired data presented higher average accuracies in correct sex classification. This is an indication that the source of data and how these data are collected may influence the outcome of the results of the analysis.

**Table 7:** Comparison of average accuracies in correct sex classification from previous studies and present study

Study	Year	Pop Gp	Data source	Measurements																								Highest average accuracy
				MAXH				MAXW				MAXT				HAF				MAFB				LAFB				
				Males		Females		Males		Females		Males		Females		Males		Females		Males		Females		Males		Females		
				Mean	SD	Mean	SD	Mean	SD	Mean	SD	Mean	SD	Mean	SD	Mean	SD	Mean	SD	Mean	SD	Mean	SD	Mean	SD	Mean	SD	
Introna et al. [68]	1998	Italian	Skeletal collection	41.2	2.9	37	2.9	43.2	2.7	39.4	3.2	20.4	1.9	18.3	1.6													63.6
Bidmos et al. [46]	2005	South Afrfican whites	Skeletal collection	43.6	3.1	38.7	3.1	45.3	3.3	40.3	3.3	20.4	1.8	18.4	1.8	30.8	2.6	27.6	3.1	20.5	1.8	18.2	2.2	28.1	2.7	25.0	2.5	85.0
Dayal and Bidmos [57]	2005	South Afrfican blacks	Skeletal collection	41.2	3.1	36.5	2.2	43.3	2.5	39.0	2.9	20.6	1.4	18.2	1.7	29.6	3.0	27.9	2.7	18.4	1.9	16.3	1.6	25.3	2.1	22.9	2.1	85
Mahfouz et al. [69]	2007	Americans	Skeletal collection, live and cadaveric	Non linear method																								93.5
Akhlagi et al. [70]	2010	Iranians	Autopsy: Caliper	44.7	2.7	38.4	2.0	45.5	2.2	40.1	1.9	21.9	1.9	20.3	1.4													92.9
Peckmann et al. [71]	2016	Spanish	Skeletal collection	42.9	3.0	37.9	3.0	44.6	3.3	4.3	2.9	20.3	1.9	18.1	1.8	25.7	2.3	23.6	2.9	19.2	2.3	17.0	1.8	24.8	2.3	22.5	2.1	84.8
Peckmann and Fischer [72]	2016	African Americans	Skeletal collection	44.8	3.5	39.8	3.3	45.0	3.8	39.8	3.3	20.8	2.0	19.2	2.0	32.9	2.3	29.6	2.6	20.8	2.2	18.0	2.1	24.4	2.2	21.5	2.3	85.0
Michiue et al. [73]	2018	Japanese	Autopsy: CT	44.1	3.5	38.8	2.7					22.5	1.5	38.8	2.7													87.7
Teke et al. [74]	2018	Turkish	MRI scans patients	41.3	3.4	35.8	2.3	46.3	3.0	40.4	3.0	22.4	1.6	19.9	1.7													89.0
Indra et al. [75]	2021	Swiss	Skeletal collection	44.2	3.1	38.7	2.5	45.3	3.3	40.3	2.9	22.7	2.1	20.3	1.7													83.8
Current study	2022	South Africans	Skeletal collection	42.1	3.1	37.3	2.8	43.8	3.3	38.8	3.2	20.4	1.6	18.1	1.8	30.3	2.7	27.4	2.8	19.7	2.0	17.5	2.2	26.3	2.7	23.4	2.6	84.2

In addition, the average accuracies for the pooled data for the patella from the current study using discriminant function analysis (81.9–84.2%) are similar to those observed for SAED (75–85%: [46]) and SAAD (78–85%: [66]). The highest drop in average accuracies in the current study (0.8%) is lower than those from population-specific DFEs for SAED and SAAD which were 2.5% and 3.3%, respectively. This observation of a lower drop of average accuracies for DFEs obtained from pooled data compared to population-specific DFEs agrees with Bidmos and Mazengenya [85] in which the highest drop in average accuracies for pooled data DFEs was 0.9%. This observation indicates a better validity of pooled DFEs compared to population-specific DFEs. Another previously documented advantage of the application of DFEs from pooled data is that they can be applied to an unknown skeleton without the prior knowledge of the population group [64].

The same performance trend is observed in the current study using the ML algorithm compared to the conventional statistical model. The standards generated for sex classification produced higher average accuracies (Table 4) compared to those generated using discriminant function analysis (Table 3). Compared to the average accuracies for the pooled data for the patella from the current study using discriminant function analysis (81.9–84.2%), the stacking machine learning approach provides an overall accuracy of 90.77%. This clearly indicates that with the application of the machine learning paradigm a better classification of sex from the patella measurement is possible.

From the aforementioned, linear and volumetric measurements of the patella are useful in human identification and have produced acceptably high average accuracies in correct sex classification. However, human identification from skeletal remains can be demanding especially in a country like South Africa with diverse population groups. Consequently, the application of population-specific DFEs in the human identification process will require the prior assignment of the population group which might be difficult if not impossible in cases where complete skeletons are not available or in the absence of bones that display obvious population-specific traits. Another confounding problem is the difficulty in the assignment of population groups to individuals who fall within the boundaries of other population groups [52]. This has led some researchers [63, 64] to propose the idea of a generation of generic standards for the estimation of sex and stature, especially for population groups that have similarities. In both studies, the authors argue for the generation and use of generic equations for sex assignment [63] and stature estimation [64] citing the lack of adequate data and bone collections from which data could be collected for the derivation of population-specific standards in some countries.

The pelvic bone is considered one of the most sexually dimorphic bones in the body based on its design for parturition in females. Measurements of this bone have been used in the generation of population-specific DFEs in different parts of the world [2]. Steyn and Patriquin [63] assessed the reliability of population-specific DFEs compared to those from pooled data from diverse population groups. They reported a comparable performance of population-specific and generic DFEs with regard to classification rates and concluded that population-specific equations are not superior to generic equations with regard to sex prediction using dimensions of the pelvic bone. Macaluso Jr [86] evaluated the reliability of generic equations that were published by Steyn and Patriquin [63] on a French sample and reported that the average accuracies of the pooled data remained unchanged when applied to a French sample. In addition, there was no significant difference between the average accuracies obtained from the use of population-specific equations and generic equations [86]. This observation provided further proof of the usefulness and applicability of generic equations for sex prediction using pelvic measurements to other related population groups, where the application of ML can significantly help.

Attempts have also been made to apply the notion of non-superiority of population-specific equations over generic equations using measurements of the vertebrae. Hora and Sládek [87] observed that anteroposterior and mediolateral body diameters were found to be universally applicable in sex prediction while other measurements of the studied vertebrae showed population specificity in the assignment of sex. In a similar study, Bidmos and Mazengenya [85] investigated the utility of pooled data in the generation of generic equations for sex prediction. They evaluated the accuracies of population-specific equations formulated from measurements of long upper limb bones of South Africans and noted that the average accuracies of generic equations are acceptably high (81 to 87%). In addition, the cross-validated accuracies remained largely unchanged thereby confirming the usefulness of these equations in cases where it becomes difficult to establish the population affinity of the skeletal remain under forensic investigation.

Recently, Indra et al. [84] assessed the validity of population-specific DFEs formulated for patella measurements of the contemporary Spanish population group on a Swiss sample. The average accuracies obtained by Indra et al. [84] ranged from 63 to 84% for patella which was similar to those presented in an earlier study by Peckmann et al. [80]. The results of the current study in which the average accuracies obtained for generic equations are comparable to those presented for population-specific equations for South Africans of European [46] and African descent [66] in agreement with the observation made in previous studies [63, 84–87]. This, therefore, shows the utility of generic equations when the patella is available for forensic analysis in South Africa.

The range of average accuracies for generic equations formulated in the current study (81.9–90.8%) is similar to those obtained for population-specific equations derived for South Africans of European (67.5–85%) and African descent (78.3–85%). This is in agreement with the observation that was made by Indra et al. [84].

## Conclusions

Prediction of sex from recovered or discovered bones in human identification is a very important step in forensic anthropologists along with the estimation of age, stature, and population affinity. In this study, we have used a dataset of 100 people collected from a sample of patella of Mixed Ancestry South Africans (MASA). Six parameters maxh, maxw, maxt, haf, lafb, and mafb were used. Two types of investigation have been carried out in this study to compare the performance of conventional statistical analysis versus the classical machine learning techniques in the estimation of sex. Different discriminant function analyses were performed for measurements that exhibited significant differences between male and female mean measurements. On the other hand, several ML algorithms were trained, validated, and tested to identify the best feature combination for detecting the sex from the patella measurements. The range of average accuracies obtained for pooled multivariate DFEs is 81.9–84.2% while the stacking ML technique provides 90.8% accuracy which compares well with those presented in previous studies. In conclusion, findings from the current study show that generic models formulated from measurements of the patella of different population groups in South Africa are useful resent with reasonably high average accuracies. Consequently, they are useful in the prediction of sex in cases when the population affinity is either difficult or impossible to ascertain and their applicability to populations of Southern Africa will require validation studies in individual populations from different countries in the region.

## Data Availability

Data available on request.
